# Human Presence in Short-Form Video Advertising: Social Judgments of Human and AI Presenters Under Privacy Concerns

**DOI:** 10.3390/bs16020240

**Published:** 2026-02-08

**Authors:** John Yang

**Affiliations:** Business School, Hankuk University of Foreign Studies, Dongdaemun-gu, Seoul 02450, Republic of Korea; johnyang@hufs.ac.kr

**Keywords:** social cognition, judgment, attribution, short-form video, human–AI communication, presentation format, responsibility, effort, trust, privacy concern

## Abstract

Digital retailing increasingly relies on short-form video advertising where human and AI presenters coexist, requiring consumers to form rapid social judgments based on minimal perceptual cues. This research examines how presentation format shapes consumer responses through perceived creator responsibility and effort, and how viewer-inferred privacy concern moderates these effects. Drawing on social cognition, deindividuation, and heuristic-cue perspectives, two online experiments (N = 656; N = 769) compared a human presenter with captions-only and with an AI avatar in retail-product video scenarios. Across both studies, the presence of a human presenter enhanced attitudes toward the video, perceived usefulness, trust, and purchase intention by sequentially increasing perceived responsibility and effort, reflecting viewers’ attributions of agency and motivational investment. Viewer-inferred privacy concern weakened these effects by attenuating responsibility attributions, demonstrating how contextual explanations recalibrate social judgments. The findings show that minimal human cues function as social cognitive signals of accountability in digital retail advertising. This research advances understanding of human judgment and decision making in consumer contexts and offers guidance for balancing human and AI communication under privacy-sensitive conditions.

## 1. Introduction

Digital retailing increasingly relies on short form video advertising in which consumers form rapid judgments about others based on minimal social cues. Social cognition research shows that humans are predisposed to infer others’ intentions, responsibility, and motivational states from perceptual signals such as faces and voices, even when information is sparse and interaction is limited (Todorov et al., 2008; Frith & Frith, 2006). These inferences allow individuals to assess accountability, sincerity, and effort within seconds, shaping subsequent evaluative and behavioral decisions.

In contemporary video advertising environments, such social cognitive processes are activated prior to deliberate evaluation of message content or product attributes. Viewers often decide whether to trust a message or consider purchase based on immediate impressions of the presenter as a social agent (Fiske & Taylor, 2013; Weiner, 2000; Shen & Wang, 2024; Li et al., 2024). Despite this, existing retail and advertising research has devoted limited attention to how minimal human cues in video presentations guide responsibility and effort attributions and how these inferences influence consumer decision making.

In the present research, anonymity and self-disclosure are conceptualized from the perspective of the content creator rather than the viewer. Specifically, a creator’s decision to appear on screen with a visible face and voice constitutes a form of self-disclosure, whereas choosing to remain visually and vocally non-disclosed constitutes functional anonymity. This creator-side self-presentation decision provides viewers with differential identifiability cues, which serve as inputs for social cognitive inference processes.

Within this environment, the present research conceptualizes the mere human presenter effect as a social cognitive inference process in which the presence of a human face or voice functions as an agentic cue. Such cues prompt viewers to attribute responsibility and effort to the content creator, independent of message quality or expertise. Social cognition and theory of mind research demonstrates that humans routinely infer internal mental states, such as intention and commitment, from minimal perceptual information (Frith & Frith, 2006; Gray et al., 2007; Westra, 2022).

Although prior advertising research shows that human cues enhance attention and engagement, these cues have largely been treated as direct persuasion signals rather than as triggers of deeper responsibility and motivation inferences. This leaves an important gap in understanding how consumers form social judgments about communicators and how these judgments guide trust and decision making in retail advertising contexts (Weiner, 2000; Shah & Oppenheimer, 2008; Hudders et al., 2021; Boerman & Müller, 2022).

Accordingly, the theoretical gap addressed in this research is not whether consumers self-disclose, but how creator self-disclosure versus creator anonymity shapes viewers’ attribution of responsibility and effort toward the communicator. This perspective extends anonymity and self-disclosure research into a creator-centered domain that has received limited attention in advertising and retail communication contexts.

Prior literature relevant to this study spans three domains: anonymity and deindividuation, self-disclosure, and source-cue effects in advertising. In the anonymity and deindividuation literature, research has examined effects on anonymous or deindividuated actors themselves, such as their reduced effort and accountability (Zimbardo, 1969; Orden et al., 1998; Shiue et al., 2010), and effects on counterparts, such as less favorable perceptions of anonymous actors’ reviews or comments (Rains, 2007; Forman et al., 2008). In the self-disclosure domain, research has examined effects on consumer responses when influencers share personal information or experiences, fostering trust, parasocial interaction, and perceived intimacy (Leite et al., 2022; Lu et al., 2023). In the source-cue literature, researchers have shown that visual and social cues in advertising, such as imagery, facial expressions, or tone, can enhance perceived authenticity, trustworthiness, and engagement by signaling social presence or production quality (Biel & Bridgwater, 1990; McQuarrie & Phillips, 2005; Sundar & Nass, 2001; Boerman & Müller, 2022). However, prior work has largely viewed such cues as direct indicators of persuasion or message credibility rather than as triggers for deeper inferences about the communicator’s underlying responsibility and effort.

Yet despite these advances, the current retail-advertising literature remains silent on how presentation format itself may elicit such inferences in video-based retail advertising. This research extends that perspective by proposing that the mere presence of a human presenter, expressed through a visible face, voice, or both, can shape perceived creator responsibility and effort, which in turn influence consumer responses. Moreover, thousands of short-form retail-product videos now prompt consumer judgments within seconds, before any interaction, self-disclosure, or influencer identity is established. This early-stage judgment context is particularly relevant in the retail video environment, where viewers often decide within moments whether to continue watching or skip the content.

This research addresses these gaps in two key ways. First, it proposes a novel sequential mediating mechanism linking presentation format to consumer responses via perceived responsibility and effort, conceptualized as social judgments involving mental state attribution about the communicator, thereby advancing both the deindividuation and self-disclosure frameworks as well as advertising heuristic theory (Kruger et al., 2004; Sundar, 2008; Shah & Oppenheimer, 2008; Metzger et al., 2010; Leite et al., 2024; De Freitas & Hafri, 2024). Second, it situates the inquiry squarely in a retail-advertising context, focusing on product video content used by retail brands or creators for product promotion and thus attending to the practical concerns of retailers and digital marketers. By doing so, the research connects social cognitive inference processes to downstream judgment and decision-making outcomes in applied consumer contexts. Accordingly, two experimental studies (N = 656; N = 769) were conducted in online retail-advertising scenarios to test the hypotheses.

## 2. Theoretical Background and Hypotheses

### 2.1. Anonymity and Deindividuation

In the present research, anonymity refers to the degree to which a content creator’s personal identity is visually or vocally concealed from viewers, resulting in reduced identifiability of the communicator. Anonymity, the concealment of an individual’s identity, has long been studied for its psychological and behavioral consequences. Classic deindividuation theory posits that anonymity reduces self-awareness and accountability, leading individuals to act in ways that deviate from personal or societal norms due to diminished responsibility (Zimbardo, 1969; Diener et al., 1980). In computer-mediated communication, anonymity similarly alters perceived responsibility and contribution levels. For example, Jessup et al. (1990) found that when individuals were less identifiable in group decision-making, their communication patterns became more detached and less accountable. Reicher et al. (1995) extended this notion with the Social Identity Model of Deindividuation (SIDE), suggesting that anonymity does not simply remove identity but can shift attention from personal to group identity, changing behavioral norms accordingly. In marketing and retail contexts, these insights imply that when content creators are less visually or vocally identifiable, audiences may attribute lower personal accountability or effort to them. This aligns with evidence that reduced identifiability often weakens perceptions of individual contribution and performance quality (Williams et al., 1981; Shiue et al., 2010).

### 2.2. Self-Disclosure

Self-disclosure refers to the sharing of personal information, experiences, or emotions with others (Bazarova & Choi, 2014). In influencer and social media marketing, higher self-disclosure typically strengthens perceived authenticity, trustworthiness, and engagement (Leite et al., 2022; Penttinen et al., 2022; Lu et al., 2023). Through self-disclosure, creators signal openness and approachability, facilitating parasocial relationships and intimacy with audiences (Forman et al., 2008; Leite et al., 2022). However, excessive or strategic self-disclosure can sometimes undermine credibility if perceived as manipulative or irrelevant (Lee & Johnson, 2022; Leite et al., 2024). Thus, disclosure effects are context-dependent and likely mediated by viewers’ inferences about the creator’s authenticity and underlying motives.

Building on these streams, the present research integrates anonymity and self-disclosure by treating a creator’s on-screen presence as a self-disclosure decision and the absence of such presence as anonymity. This integration provides a direct theoretical pathway to responsibility and effort attributions. When creators are visually and vocally disclosed, viewers receive stronger identifiability cues, which trigger attributions of agency, accountability, and motivational investment. When creators remain non-disclosed, these attributions are weakened. This mechanism forms the basis for the proposed serial mediation linking presentation format, perceived creator responsibility, perceived creator effort, and viewer responses.

### 2.3. Human Presenter Presence, Perceived Creator Responsibility, and Effort

Perceived creator responsibility is defined as the extent to which viewers believe that the content creator feels accountable for and personally stands behind the presented message. From a social cognitive perspective, identifiability cues such as faces and voices enhance perceptions of agency, which in turn strengthen responsibility attributions. Research on mind perception shows that observers are more likely to assign intention, moral responsibility, and effort to targets perceived as agentic social actors (Gray et al., 2007; Waytz et al., 2010; De Freitas & Hafri, 2024). When such cues are absent, perceived agency declines, leading viewers to discount personal accountability and motivational investment in the content.

From the viewer’s perspective, a visible human presenter may therefore act as a responsibility signal, suggesting that the creator personally endorses and stands behind the message. This signal interpretation aligns with signaling theory (Spence, 1978), which posits that observable cues convey unobservable qualities such as credibility, commitment, or effort (Kirmani & Rao, 2000). Consequently, the presence of a human presenter can lead viewers to infer that the creator has invested greater time, attention, and energy into producing the content, enhancing perceived effort.

Perceived creator effort is defined as the extent to which viewers infer that the creator invested time, energy, and motivational commitment in producing the content. Such inferred effort operates as a heuristic for evaluating sincerity and content quality (Kruger et al., 2004; Shah & Oppenheimer, 2008), which in turn strengthens perceived trust and value (Homer, 1995; Modig et al., 2014; Chan et al., 2017). Recent marketing research further shows that perceived human versus machine agency systematically shapes trust and evaluative responses toward communicators, even when message content is held constant (Longoni & Cian, 2022). This provides converging evidence that social judgments about communicators play a central role in contemporary technology-mediated persuasion environments.

In the advertising context, where consumers rely heavily on quick visual impressions, these inferences may have amplified effects on downstream outcomes such as attitude toward the ad, perceived usefulness, trust, and purchase intention, which are collectively conceptualized as integrated viewer responses. Taken together, anonymity and self-disclosure operate in this research as creator-side identity signals that initiate social cognitive inference processes. These inferences directly map onto the hypothesized mediation structure in which presentation format influences responsibility attributions, which shape effort perceptions, which subsequently guide evaluative and behavioral responses. Accordingly, the following hypothesis is proposed:

**H1:**  *The presence of a human presenter enhances integrated viewer responses through increased viewer-perceived creator responsibility and effort in sequence.*

### 2.4. Human Presenter Presence and Privacy Concern

Privacy concern refers to apprehension about how personal information is collected and used, reflecting perceptions of risk and lack of control (Smith et al., 1996). Privacy-calculus research suggests that individuals assess the trade-off between disclosure benefits and potential privacy risks when deciding whether or not to reveal personal information (Culnan & Armstrong, 1999; Dinev & Hart, 2006; Kehr et al., 2015). Viewer inferred privacy concern introduces a contextual explanation that reshapes social cognitive interpretations of non-disclosure. Attribution research demonstrates that providing situational explanations can alter responsibility judgments by shifting perceived motives behind an action (Kelley, 1973; Weiner, 2000). Accordingly, when non-disclosure is interpreted as a privacy protection strategy rather than low involvement, default responsibility and effort attributions toward the creator are attenuated. Recent marketing research further emphasizes that privacy concerns play a central role in shaping trust and evaluative responses in digital communication environments (Martin & Murphy, 2017).

In digital retail environments, where creators increasingly act as micro-retailers or brand partners, maintaining a balance between personal privacy and authenticity is essential. Consequently, viewer-inferred privacy concern becomes a critical moderating factor that may reshape attributions of responsibility and effort. Thus, the following hypothesis is proposed:

**H2:**  *Viewer-inferred privacy concern weakens the positive effect of human presenter presence on perceived creator responsibility, thereby attenuating the downstream impact on perceived creator effort and integrated viewer responses.*

**Figure 1:**
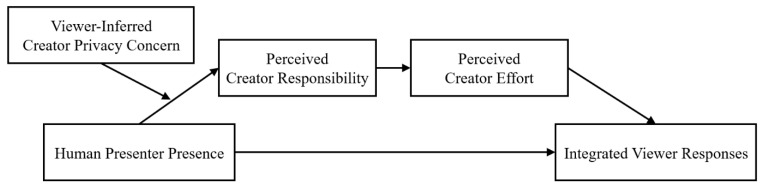
Moderated serial mediation model.

See Figure 1 for the conceptual model illustrating these hypothesized relationships. The present research comprises two studies, each of which simultaneously tests H1 and H2. Study 1 compares a human presenter (featuring face, voice, and captions) with a captions-only condition, whereas Study 2 contrasts a human presenter with an AI presenter (featuring AI-generated face and voice).

## 3. Study 1

Study 1 tested H1 and H2 by comparing a video featuring a human presenter (face, voice, and captions) with one using only captions, examining differences in perceived creator responsibility, perceived creator effort, and integrated viewer responses, as well as the moderating role of viewer-inferred privacy concern.

### 3.1. Method

A total of 701 participants were recruited from Prolific and received £0.30 for completing the survey. Participants identified as potential bots via Google reCAPTCHA (Qualtrics, 2024) were excluded, leaving 656 valid participants for analysis.

Study 1 employed an experimental between-subjects design with randomized assignment to manipulated presentation-format conditions. Participants were randomly assigned to one of four conditions in a 2 (presentation format: human presenter present vs. absent) × 2 (inferred creator privacy concern: high vs. low) between-subjects design. In the human-presenter condition, participants read a scenario describing a social-media product-review video in which the creator appeared on screen, explained the product verbally, and used captions. In the presenter-absent condition, the scenario stated that the creator did not appear or speak, and product information was presented only through captions. No specific product, brand, or product category was mentioned or shown in the scenario. The description focused solely on the creator’s presentation format to avoid confounding effects of product type. To manipulate inferred creator privacy concern, participants in the high-privacy-concern condition were informed that many creators avoid showing their face or voice to protect privacy, and even those who do so are aware that it may involve a privacy cost. The low-privacy-concern condition omitted this explanation.

Following the scenario, participants rated inferred creator privacy concern, perceived creator responsibility, perceived creator effort, and integrated viewer responses, comprising attitude toward the video, perceived usefulness, trust, and purchase intention toward the featured product. The product category was intentionally left unspecified to prevent product-specific bias. All items were measured on 7-point Likert-type scales (1 = strongly disagree, 7 = strongly agree).

Viewer inferred privacy concern was measured with five items (α = 0.95): “Creators feel a strong fear of disclosing their personal information,” “creators are strongly concerned about the privacy of the personal information they disclose in their videos,” “creators are very much concerned that the personal information they share could be misused,” “creators feel really uneasy about sharing personal information in their videos, because of how others might use it,” and “creators strongly worry that the personal information disclosed could be used in unexpected ways” (Xu et al., 2011a; Dolnicar & Jordaan, 2007).

Viewer perceived creator responsibility was measured with three items (α = 0.94): “I think the creator feels a strong sense of responsibility for the video,” “I think the creator feels a strong sense of accountability for the video,” and “I think the creator feels a strong sense of obligation regarding the video” (Peck et al., 2021).

Viewer perceived creator effort was measured with four items (α = 0.95): “The creator was fully committed to this video,” “the creator tried their hardest to create this video,” “the creator devoted a huge amount of energy and attention to creating this video,” and “the creator put a lot of time and effort into making this video” (Rich et al., 2010).

Attitude toward the video was measured with six items (α = 0.97): “I think this video would be very impactful,” “I think this video would be very good,” “I think this video would be very appealing,” “I think this video would be highly effective,” “I really like this video,” and “overall, my impression of the video is very positive” (Bruner & Kumar, 2000; Olsen & Pracejus, 2020).

Perceived usefulness of video was measured with three items (α = 0.95): “I think this video can provide very useful information about the product,” “I think this video can really help viewers better understand the product,” and “overall, I think this video can be very useful” (Ali et al., 2023).

Trust in the video was measured with five items (α = 0.98): “I think this video would be very trustworthy,” “I think this video would be highly reliable,” “I think this would be a really honest video,” “I think this video would be very credible,” and “I think this video would be very truthful” (Olsen & Pracejus, 2020; Chaudhuri & Holbrook, 2001).

Purchase intention was measured with two items (α = 0.96): “If I needed this kind of product, I would choose the one shown in this video over those from other brands” and “if I needed this kind of product, I would buy the one shown in this video instead of buying from another brand” (Chaudhuri & Holbrook, 2001).

### 3.2. Results

A manipulation check confirmed that the privacy manipulation worked as intended. Participants in the high-privacy-concern condition reported significantly greater inferred privacy concern than those in the low-privacy-concern condition (*M*’s = 4.95 vs. 4.04; *F*(1, 654) = 62.30, *p* < 0.001, η^2^ = 0.087).

Human presenter presence significantly increased perceived creator responsibility (*M*’s = 5.40 vs. 4.78; *F*(1, 654) = 35.78, *p* < 0.001, η^2^ = 0.052) and perceived creator effort (*M*’s = 5.45 vs. 4.62; *F*(1, 654) = 71.41, *p* < 0.001, η^2^ = 0.098). It also enhanced all four components of integrated viewer responses: attitude toward the video (*M*’s = 4.60 vs. 3.96; *F*(1, 654) = 34.32, *p* < 0.001, η^2^ = 0.050), perceived usefulness (*M*’s = 5.14 vs. 4.85; *F*(1, 654) = 8.93, *p* = 0.003, η^2^ = 0.013), trust (*M*’s = 4.53 vs. 4.35; *F*(1, 654) = 2.77, *p* = 0.097, η^2^ = 0.004), and purchase intention (*M*’s = 4.21 vs. 3.75; *F*(1, 654) = 18.87, *p* < 0.001, η^2^ = 0.028).

Two complementary analyses were conducted to test distinct hypotheses. PROCESS Model 6 was used to examine the baseline serial mediation mechanism proposed in H1. PROCESS Model 83 was then used to test whether this serial mediation process was conditionally moderated by viewer-inferred privacy concern as proposed in H2.

To test the hypothesized sequential mediation, four serial mediation analyses were conducted using PROCESS Model 6 (Hayes, 2017; 10,000 bootstrap samples). Results revealed significant positive indirect effects of human-presenter presence on all four dependent variables via perceived creator responsibility and perceived creator effort in sequence: attitude toward the video (b = 0.25, 95% CI [0.1566, 0.3571]); perceived usefulness (b = 0.18, 95% CI [0.1047, 0.2626]); trust (b = 0.14, 95% CI [0.0731, 0.2296]); and purchase intention (b = 0.15, 95% CI [0.0741, 0.2414]). These findings support H1, indicating that the presence of a human presenter enhances integrated viewer responses by sequentially increasing perceived creator responsibility and effort.

Finally, moderated serial mediation analyses using PROCESS Model 83 (Hayes, 2017; 10,000 bootstrap samples) showed that the indirect effects were conditional on viewer-inferred privacy concern. The path from human presenter presence to perceived creator responsibility was significantly moderated by inferred privacy concern, such that the positive effect was weaker when viewers perceived higher privacy concerns. Results indicate that the serial indirect effect of presenter presence through perceived responsibility and effort is positive and significant. The negative coefficients reported in the moderated mediation analysis reflect attenuation of this positive indirect effect as viewer-inferred privacy concern increases, rather than a reversal of the mediation direction. Conditional indirect effects of human-presenter presence were significant for all four dependent variables: attitude toward the video (b = −0.07, 95% CI [−0.1254, −0.0119]); perceived usefulness (b = −0.05, 95% CI [−0.0882, −0.0084]); trust (b = −0.04, 95% CI [−0.0783, −0.0066]); and purchase intention (b = −0.04, 95% CI [−0.0816, −0.0054]). These results support H2, demonstrating that inferred privacy concern attenuates the positive impact of human-presenter presence on perceived creator responsibility and its downstream effects on perceived effort and integrated viewer responses.

## 4. Study 2

Study 2 was designed to replicate and extend the findings of Study 1 by testing H1 and H2 in a human-versus-AI presenter context. Whereas Study 1 examined the effect of human presence relative to the absence of a visible or audible presenter, Study 2 serves a distinct theoretical purpose by isolating whether responsibility and effort attributions depend on human agency itself rather than on the mere presence of a speaking or animated entity. Specifically, the study compared a video featuring a human presenter (face and voice) with one featuring an AI avatar (AI-generated face and voice), examining differences in perceived creator responsibility, perceived creator effort, and integrated viewer responses, as well as the moderating role of viewer-inferred privacy concern.

By contrasting a human presenter with an AI-generated social actor that provides comparable visual and vocal cues, Study 2 allows for a stronger test of social cognitive inferences about agency, responsibility, and motivational investment. This design rules out alternative explanations based on audiovisual richness or presentation clarity and directly assesses whether human-specific agency cues drive responsibility and effort attributions.

### 4.1. Method

A total of 805 participants from Prolific completed the survey and received £0.30 as compensation. Participants identified as bots through Google reCAPTCHA (Qualtrics, 2024) were excluded, leaving 769 valid responses for analysis.

**Figure 2:**
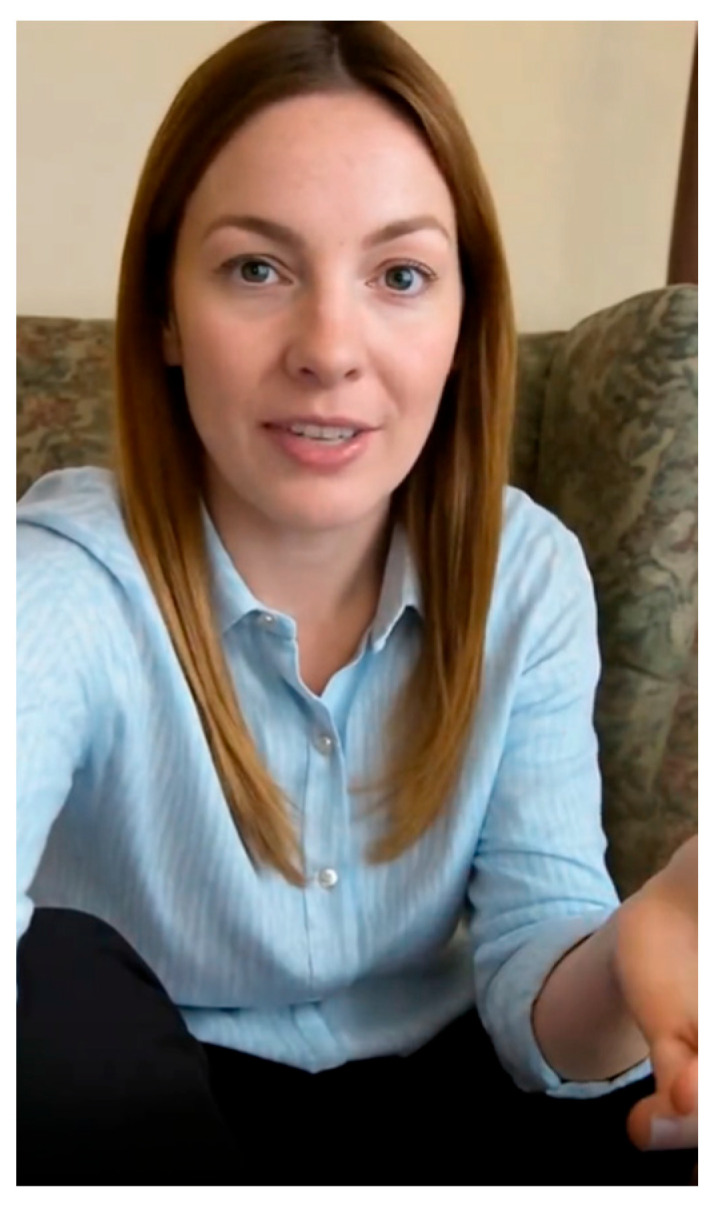
The screenshot of the video provided in Study 2.

The procedure was identical to Study 1 except that the presenter was either a human or an AI avatar. Participants were randomly assigned to one of four conditions in a 2 (presentation format: human vs. AI) × 2 (inferred creator privacy concern: high vs. low) between-subjects design. In the human-presenter condition, the scenario stated that the creator appeared on screen and explained the product. In the AI-presenter condition, the creator was absent; instead, a virtual AI avatar presented the product review using an AI-generated voice. All participants then viewed the same screenshot from the video (see Figure 2), taken from a clip produced with Topview, an AI video-production tool. The screenshot displayed only the presenter’s upper body and did not include any visible product, brand, or category information, ensuring that evaluations were not influenced by product-related cues.

All measures and scales were identical to those used in Study 1, including inferred creator privacy concern, perceived creator responsibility, perceived creator effort, attitude toward the video, perceived usefulness, trust, and purchase intention. Responses were recorded on 7-point Likert-type scales (1 = strongly disagree, 7 = strongly agree).

### 4.2. Results

A manipulation check confirmed that participants in the human-presenter condition perceived the creator as a real person significantly more than those in the AI-presenter condition (*M*’s = 5.05 vs. 2.75; *F*(1, 767) = 329.99, *p* < 0.001, η^2^ = 0.301). The privacy-concern manipulation check confirmed that participants in the high-privacy-concern condition reported significantly greater inferred privacy concern than those in the low-concern condition (*M*’s = 4.88 vs. 4.36; *F*(1, 767) = 23.53, *p* < 0.001, η^2^ = 0.030).

Human presenter presence again produced significant effects on key variables. Participants who viewed a human presenter perceived greater creator responsibility (*M*’s = 5.24 vs. 4.52; *F*(1, 767) = 49.25, *p* < 0.001, η^2^ = 0.060) and effort (*M*’s = 5.08 vs. 4.18; *F*(1, 767) = 76.18, *p* < 0.001, η^2^ = 0.090) than those who viewed an AI presenter. Similarly, the human presenter condition yielded more favorable evaluations across the integrated viewer responses: attitude toward the video (*M*’s = 4.31 vs. 3.50; *F*(1, 767) = 55.91, *p* < 0.001, η^2^ = 0.068), perceived usefulness (*M*’s = 4.88 vs. 4.43; *F*(1, 767) = 19.65, *p* < 0.001, η^2^ = 0.025), trust (*M*’s = 4.63 vs. 3.78; *F*(1, 767) = 68.31, *p* < 0.001, η^2^ = 0.082), and purchase intention (*M*’s = 4.21 vs. 3.75; *F*(1, 767) = 48.48, *p* < 0.001, η^2^ = 0.059).

Two complementary analyses were conducted to test distinct hypotheses. PROCESS Model 6 was used to examine the baseline serial mediation mechanism proposed in H1. PROCESS Model 83 was then used to test whether this serial mediation process was conditionally moderated by viewer-inferred privacy concern as proposed in H2.

Serial mediation analyses using PROCESS Model 6 (Hayes, 2017; 10,000 bootstrap samples), controlling for age and gender, revealed significant positive indirect effects of human presenter presence on all four dependent variables through perceived creator responsibility and perceived creator effort in sequence: attitude toward the video (b = 0.33, 95% CI [0.2304, 0.4473]); perceived usefulness (b = 0.23, 95% CI [0.1503, 0.3170]); trust (b = 0.26, 95% CI [0.1754, 0.3571]); and purchase intention (b = 0.24, 95% CI [0.1597, 0.3397]). These results support H1, confirming that human presenter presence enhances integrated viewer responses via sequential increases in perceived responsibility and effort.

Finally, moderated serial mediation analyses using PROCESS Model 83 (Hayes, 2017; 10,000 bootstrap samples) showed significant conditional indirect effects on all four dependent variables, again with the path from presenter presence to perceived responsibility moderated by viewer-inferred privacy concern. The positive effect of human presenter presence on perceived responsibility was weaker when viewers inferred greater privacy concern: attitude toward the video (b = −0.07, 95% CI [−0.1437, −0.0021]); perceived usefulness (b = −0.05, 95% CI [−0.1007, −0.0019]); trust (b = −0.06, 95% CI [−0.1145, −0.0019]); and purchase intention (b = −0.05, 95% CI [−0.1086, −0.0017]). Results indicate that the serial indirect effect of presenter presence through perceived responsibility and effort is positive and significant. The negative coefficients reported in the moderated mediation analysis reflect attenuation of this positive indirect effect as viewer-inferred privacy concern increases, rather than a reversal of the mediation direction. These findings support H2, demonstrating that viewer-inferred privacy concern attenuates the positive impact of human presenter presence on perceived responsibility and its downstream effects on perceived effort and integrated viewer responses. Together with Study 1, these results indicate that the proposed serial mediation and its moderation by viewer-inferred privacy concern are robust across different creator self-disclosure conditions, including non-disclosed human-presenter settings and AI-presenter settings, reinforcing the generalizability of the underlying social judgment mechanism. Table 1 provides a summary of the serial mediation and moderated mediation results across Study 1 and Study 2.

## 5. General Discussion

This research provides an integrative explanation of how presentation format in video advertising shapes consumer responses and when this effect varies. Across two experiments, the presence of a human presenter consistently enhanced consumer evaluations by increasing perceived creator responsibility and effort, while viewer-inferred privacy concern moderated these effects, shaping how human and AI presenters are evaluated under privacy-sensitive conditions. The findings show that even minimal human cues can function as social cognitive signals of agency, accountability, and authenticity, influencing how viewers form judgments and make evaluative decisions in an AI-driven retail environment.

Study 1 tests the proposed mechanism by comparing disclosed versus non-disclosed human presenter conditions, examining how creator self-presentation shapes responsibility and effort attributions and how viewer-inferred privacy concern attenuates this process. Study 2 extends this mechanism by contrasting a disclosed human presenter with an AI-presenter condition in which the human creator is non-disclosed, testing whether responsibility and effort attributions depend on creator self-disclosure rather than audiovisual presentation alone, again under varying levels of viewer-inferred privacy concern. The consistency of both the serial mediation and its moderation by privacy concern across the two studies indicates that responsibility and effort attributions are driven by creator self-disclosure decisions, while contextual privacy inferences recalibrate these social judgments even when AI-generated presenters provide comparable visual and vocal cues. By linking identifiability, effort perception, and privacy inference to persuasion, this study clarifies how consumers form judgments in digital retail contexts and offers guidance for balancing human and AI communication.

### 5.1. Theoretical Contributions

This research contributes to social cognition literature by demonstrating how minimal perceptual cues in video advertising trigger systematic inferences about responsibility and effort, which subsequently guide judgment and decision making. By showing that faces and voices function as agentic signals rather than mere attention cues, the findings extend face perception and theory of mind research into consumer contexts (Todorov et al., 2008; Gray et al., 2007). Moreover, identifying viewer inferred privacy concern as a moderator highlights how contextual framing can recalibrate social judgments and mitigate attributional bias (Kelley, 1973; Weiner, 2000). Demonstrating that human presence through face or voice heightens perceived responsibility, effort, and evaluation, this research extends heuristic cue theory (Shah & Oppenheimer, 2008) and attribution theory (Weiner, 2000) to digital advertising contexts.

Building on this, the findings demonstrate that responsibility and effort attributions operate as distinct social-cognitive mechanisms linking identifiability cues to evaluative and behavioral responses, rather than merely reflecting general engagement or message credibility. This refines deindividuation and self-disclosure frameworks by specifying the precise attributional pathway through which creator disclosure decisions shape consumer judgment in short-form video advertising environments.

Moreover, this research integrates deindividuation (Zimbardo, 1969) and self-disclosure research (Joinson, 2001; Lee & Johnson, 2022) by identifying identifiability as the bridge between anonymity and engagement. Earlier studies examined these domains separately, whereas this work shows that even minimal identifiability can evoke responsibility and effort attributions. Incorporating viewer-inferred privacy concern as a moderator further specifies boundary conditions consistent with privacy-calculus theory (Culnan & Armstrong, 1999; Xu et al., 2011b). Collectively, the findings refine digital persuasion and social presence perspectives (Sundar, 2008; Ghazali et al., 2018) by revealing a cognitive and motivational route through which presentation format shapes consumer judgment in retail advertising. By demonstrating that both the serial mediation mechanism and its moderation by viewer-inferred privacy concern persist across disclosed and non-disclosed creator conditions and across AI-presenter contexts, this research extends social cognition and attribution theory into AI-mediated advertising environments and clarifies how contextual privacy inferences recalibrate early-stage social judgments.

### 5.2. Managerial Implications

The findings provide clear implications for retailers and marketers designing short-form video content. Featuring a human presenter, whether a brand representative or an independent creator, enhances consumer evaluations even when the message and visuals are standardized by increasing perceived accountability and effort, which are key drivers of credibility and trust (Boerman & Müller, 2022; Eisend, 2006). Consequently, retailers with limited sources can prioritize presenter format over expensive production features to strengthen advertising effectiveness.

When human presenters cannot or prefer not to disclose their identity due to privacy concerns, AI-presenter formats offer a scalable alternative for short-form video advertising. However, replacing a disclosed human presenter with an AI presenter introduces a non-disclosed creator condition, which may weaken responsibility and effort attributions unless appropriate contextual cues are provided. Firms can mitigate this risk by explicitly communicating that AI presenters are used to protect creator privacy, thereby preserving perceived accountability while maintaining production efficiency. These findings provide practical guidance for deploying AI-generated presenters while balancing privacy protection and persuasive effectiveness in digital advertising environments.

### 5.3. Limitations and Future Research

This research has limitations that suggest opportunities for future inquiry. First, the studies used scenario-based online experiments, which offer internal validity but may not fully capture real consumer behavior. Future research could validate these findings in field settings or with behavioral indicators such as viewing time, click-throughs, or purchase data (Batra & Keller, 2016). Second, while this study focused on short-form videos, similar mechanisms may operate in livestreaming or influencer commerce contexts (Wongkitrungrueng & Assarut, 2020), where real-time interaction and parasocial cues intensify perceptions of responsibility. Relatedly, future research could further connect these social cognitive mechanisms to responsibility-based processes identified in AI-powered advertising research (Yang et al., 2025), particularly by examining how responsibility attributions shape downstream consumer judgments and behavioral intentions across different advertising technologies. Third, future work could explore boundary conditions such as product category, platform norms, or cultural variations in self-presentation, which may shape how viewers infer responsibility and effort (Markus & Kitayama, 1991). Finally, in Study 2, the human-presenter condition featured a female-presenting figure in the screenshot stimulus. Although no individuating information was provided and presentation format was held constant across conditions, future research could systematically vary presenter gender and other visual attributes to examine whether such cues further shape responsibility and effort attributions.

## Figures and Tables

**Table 1:** Summary of serial mediation and moderated mediation results across Study 1 and Study 2.

Hypothesized Relationship	Study 1 Effect (b, 95% CI)	Study 2 Effect (b, 95% CI)	Supported
H1: Serial mediation (Attitude)	0.25 [0.1566, 0.3571]	0.33 [0.2304, 0.4473]	Yes
H1: Serial mediation (Usefulness)	0.18 [0.1047, 0.2626]	0.23 [0.1503, 0.3170]	Yes
H1: Serial mediation (Trust)	0.14 [0.0731, 0.2296]	0.26 [0.1754, 0.3571]	Yes
H1: Serial mediation (Purchase intention)	0.15 [0.0741, 0.2414]	0.24 [0.1597, 0.3397]	Yes
H2: Moderation (conditional indirect effect on Attitude)	−0.07 [−0.1254, −0.0119]	−0.07 [−0.1437, −0.0021]	Yes
H2: Moderation (conditional indirect effect on Usefulness)	−0.05 [−0.0882, −0.0084]	−0.05 [−0.1007, −0.0019]	Yes
H2: Moderation (conditional indirect effect on Trust)	−0.04 [−0.0783, −0.0066]	−0.06 [−0.1145, −0.0019]	Yes
H2: Moderation (conditional indirect effect on Purchase intention)	−0.04 [−0.0816, −0.0054]	−0.05 [−0.1086, −0.0017]	Yes

## Data Availability

The data presented in this study are available on request from the corresponding author due to privacy and ethical restrictions.

## References

[B1-behavsci-16-00240] Ali F., Yasar B., Ali L., Dogan S. (2023). Antecedents and consequences of travelers’ trust towards personalized travel recommendations offered by ChatGPT. International Journal of Hospitality Management.

[B2-behavsci-16-00240] Batra R., Keller K. L. (2016). Integrating marketing communications: New findings, new lessons, and new ideas. Journal of Marketing.

[B3-behavsci-16-00240] Bazarova N. N., Choi Y. H. (2014). Self-disclosure in social media: Extending the functional approach to disclosure motivations and characteristics on social network sites. Journal of Communication.

[B4-behavsci-16-00240] Biel A. L., Bridgwater C. A. (1990). Attributes of likable television commercials. Journal of Advertising Research.

[B5-behavsci-16-00240] Boerman S. C., Müller C. M. (2022). Understanding which cues people use to identify influencer marketing on Instagram: An eye tracking study and experiment. International Journal of Advertising.

[B6-behavsci-16-00240] Bruner G. C., Kumar A. (2000). Web commercials and advertising hierarchy-of-effects. Journal of Advertising Research.

[B7-behavsci-16-00240] Chan F. Y., Chan H. F., Tang F. (2017). The effect of perceived advertising effort on brand perception: Implication for retailers in Hong Kong. The International Review of Retail, Distribution and Consumer Research.

[B8-behavsci-16-00240] Chaudhuri A., Holbrook M. B. (2001). The chain of effects from brand trust and brand affect to brand performance: The role of brand loyalty. Journal of Marketing.

[B9-behavsci-16-00240] Culnan M. J., Armstrong P. K. (1999). Information privacy concerns, procedural fairness, and impersonal trust: An empirical investigation. Organization Science.

[B10-behavsci-16-00240] De Freitas J., Hafri A. (2024). Moral thin-slicing: Forming moral impressions from a brief glance. Journal of Experimental Social Psychology.

[B11-behavsci-16-00240] Diener E., Lusk R., DeFour D., Flax R. (1980). Deindividuation: Effects of group size, density, number of observers, and group member similarity on self-consciousness and disinhibited behavior. Journal of Personality and Social Psychology.

[B12-behavsci-16-00240] Dinev T., Hart P. (2006). An extended privacy calculus model for e-commerce transactions. Information Systems Research.

[B13-behavsci-16-00240] Dolnicar S., Jordaan Y. (2007). A market-oriented approach to responsibly managing information privacy concerns in direct marketing. Journal of Advertising.

[B14-behavsci-16-00240] Eisend M. (2006). Source credibility dimensions in marketing communication—A generalized solution. Journal of Empirical Generalisations in Marketing Science.

[B15-behavsci-16-00240] Fiske S. T. T., Taylor S. E. (2013). Social cognition: From brains to culture.

[B16-behavsci-16-00240] Forman C., Ghose A., Wiesenfeld B. (2008). Examining the relationship between reviews and sales: The role of reviewer identity disclosure in electronic markets. Information Systems Research.

[B17-behavsci-16-00240] Frith C. D., Frith U. (2006). The neural basis of mentalizing. Neuron.

[B18-behavsci-16-00240] Ghazali A. S., Ham J., Barakova E., Markopoulos P. (2018). The influence of social cues in persuasive social robots on psychological reactance and compliance. Computers in Human Behavior.

[B19-behavsci-16-00240] Gray H. M., Gray K., Wegner D. M. (2007). Dimensions of mind perception. Science.

[B20-behavsci-16-00240] Hayes A. F. (2017). Introduction to mediation, moderation, and conditional process analysis: A regression-based approach.

[B21-behavsci-16-00240] Homer P. M. (1995). Ad size as an indicator of perceived advertising costs and effort: The effects on memory and perceptions. Journal of Advertising.

[B22-behavsci-16-00240] Hudders L., De Jans S., De Veirman M. (2021). The commercialization of social media stars: A literature review and conceptual framework on the strategic use of social media influencers. Social media influencers in strategic communication.

[B23-behavsci-16-00240] Jessup L. M., Connolly T., Tansik D. A. (1990). Toward Atheory of automated group work: The deindividuating effects of anonymity. Small group research.

[B24-behavsci-16-00240] Joinson A. N. (2001). Self-disclosure in computer-mediated communication: The role of self-awareness and visual anonymity. European Journal of Social Psychology.

[B25-behavsci-16-00240] Kehr F., Kowatsch T., Wentzel D., Fleisch E. (2015). Blissfully ignorant: The effects of general privacy concerns, general institutional trust, and affect in the privacy calculus. Information Systems Journal.

[B26-behavsci-16-00240] Kelley H. H. (1973). The processes of causal attribution. American Psychologist.

[B27-behavsci-16-00240] Kirmani A., Rao A. R. (2000). No pain, no gain: A critical review of the literature on signaling unobservable product quality. Journal of Marketing.

[B28-behavsci-16-00240] Kruger J., Wirtz D., Van Boven L., Altermatt T. W. (2004). The effort heuristic. Journal of Experimental Social Psychology.

[B29-behavsci-16-00240] Lee S. S., Johnson B. K. (2022). Are they being authentic? The effects of self-disclosure and message sidedness on sponsored post effectiveness. International Journal of Advertising.

[B30-behavsci-16-00240] Leite F. P., Pontes N., de Paula Baptista P. (2022). Oops, I’ve overshared! When social media influencers’ self-disclosure damage perceptions of source credibility. Computers in Human Behavior.

[B31-behavsci-16-00240] Leite F. P., Pontes N., Septianto F. (2024). To share or not to share: When is influencer self-disclosure perceived as appropriate?. Journal of Consumer Behaviour.

[B32-behavsci-16-00240] Li N., Xuan C., Chen R. (2024). Different roles of two kinds of digital coexistence: The impact of social presence on consumers’ purchase intention in the live streaming shopping context. Journal of Retailing and Consumer Services.

[B33-behavsci-16-00240] Longoni C., Cian L. (2022). Artificial intelligence in utilitarian vs. hedonic contexts: The “word-of-machine” effect. Journal of Marketing.

[B34-behavsci-16-00240] Lu Y., Liu X., Hu Y., Zhu C. (2023). Influence of livestreamers’ intimate self-disclosure on tourist responses: The lens of parasocial interaction theory. Journal of Hospitality and Tourism Management.

[B35-behavsci-16-00240] Markus H. R., Kitayama S. (1991). Cultural variation in the self-concept. The self: Interdisciplinary approaches.

[B36-behavsci-16-00240] Martin K. D., Murphy P. E. (2017). The role of data privacy in marketing. Journal of the Academy of Marketing Science.

[B37-behavsci-16-00240] McQuarrie E. F., Phillips B. J. (2005). Indirect persuasion in advertising: How consumers process metaphors presented in pictures and words. Journal of Advertising.

[B38-behavsci-16-00240] Metzger M. J., Flanagin A. J., Medders R. B. (2010). Social and heuristic approaches to credibility evaluation online. Journal of Communication.

[B39-behavsci-16-00240] Modig E., Dahlén M., Colliander J. (2014). Consumer-perceived signals of ‘creative’ versus ‘efficient’ advertising: Investigating the roles of expense and effort. International Journal of Advertising.

[B40-behavsci-16-00240] Olsen G. D., Pracejus J. W. (2020). Customized advertising: Allowing consumers to directly tailor messages leads to better outcomes for the brand. Journal of Business Research.

[B41-behavsci-16-00240] Orden H. V., Claudia Y. D., Gaillard A. W., Buunk B. P. (1998). Social loafing under fatigue. Journal of Personality and Social Psychology.

[B42-behavsci-16-00240] Peck J., Kirk C. P., Luangrath A. W., Shu S. B. (2021). Caring for the commons: Using psychological ownership to enhance stewardship behavior for public goods. Journal of Marketing.

[B43-behavsci-16-00240] Penttinen V., Ciuchita R., Čaić M. (2022). YouTube it before you buy it: The role of parasocial interaction in consumer-to-consumer video reviews. Journal of Interactive Marketing.

[B44-behavsci-16-00240] Qualtrics (2024). Response quality. Qualtrics XM: The leading experience management software.

[B45-behavsci-16-00240] Rains S. A. (2007). The impact of anonymity on perceptions of source credibility and influence in computer-mediated group communication: A test of two competing hypotheses. Communication Research.

[B46-behavsci-16-00240] Reicher S. D., Spears R., Postmes T. (1995). A social identity model of deindividuation phenomena. European Review of Social Psychology.

[B47-behavsci-16-00240] Rich B. L., Lepine J. A., Crawford E. R. (2010). Job engagement: Antecedents and effects on job performance. Academy of Management Journal.

[B48-behavsci-16-00240] Shah A. K., Oppenheimer D. M. (2008). Heuristics made easy: An effort-reduction framework. Psychological Bulletin.

[B49-behavsci-16-00240] Shen X., Wang J. (2024). How short video marketing influences purchase intention in social commerce: The role of users’ persona perception, shared values, and individual-level factors. Humanities and Social Sciences Communications.

[B50-behavsci-16-00240] Shiue Y. C., Chiu C. M., Chang C. C. (2010). Exploring and mitigating social loafing in online communities. Computers in Human Behavior.

[B51-behavsci-16-00240] Smith H. J., Milberg S. J., Burke S. J. (1996). Information privacy: Measuring individuals’ concerns about organizational practices. MIS Quarterly.

[B52-behavsci-16-00240] Spence M. (1978). Job market signaling. Uncertainty in economics.

[B53-behavsci-16-00240] Sundar S. S. (2008). The MAIN model: A heuristic approach to understanding technology effects on credibility.

[B54-behavsci-16-00240] Sundar S. S., Nass C. (2001). Conceptualizing sources in online news. Journal of Communication.

[B55-behavsci-16-00240] Todorov A., Said C. P., Engell A. D., Oosterhof N. N. (2008). Understanding evaluation of faces on social dimensions. Trends in Cognitive Sciences.

[B56-behavsci-16-00240] Waytz A., Gray K., Epley N., Wegner D. M. (2010). Causes and consequences of mind perception. Trends in Cognitive Sciences.

[B57-behavsci-16-00240] Weiner B. (2000). Attributional thoughts about consumer behavior. Journal of Consumer Research.

[B58-behavsci-16-00240] Westra E. (2022). Social cognition and theory of mind. Mind, cognition, and neuroscience.

[B59-behavsci-16-00240] Williams K., Harkins S. G., Latané B. (1981). Identifiability as a deterrant to social loafing: Two cheering experiments. Journal of Personality and Social Psychology.

[B60-behavsci-16-00240] Wongkitrungrueng A., Assarut N. (2020). The role of live streaming in building consumer trust and engagement with social commerce sellers. Journal of Business Research.

[B61-behavsci-16-00240] Xu H., Dinev T., Smith J., Hart P. (2011a). Information privacy concerns: Linking individual perceptions with institutional privacy assurances. Journal of the Association for Information Systems.

[B62-behavsci-16-00240] Xu H., Luo X. R., Carroll J. M., Rosson M. B. (2011b). The personalization privacy paradox: An exploratory study of decision making process for location-aware marketing. Decision Support Systems.

[B63-behavsci-16-00240] Yang J., Maeng A., Jung S. U. (2025). The impact of AI-powered Ad customization: Exploring the impact of engagement, psychological ownership, responsibility, and attitude on behavioral intentions. Journal of Advertising.

[B64-behavsci-16-00240] Zimbardo P. G. (1969). The human choice: Individuation, reason, and order versus deindividuation, impulse, and chaos. Nebraska symposium on motivation.

